# Preconcentration of rifampicin prior to its efficient spectroscopic determination in the wastewater samples based on a nonionic surfactant

**DOI:** 10.3906/kim-2102-28

**Published:** 2021-08-27

**Authors:** Afaq Ullah KHAN, Faheem SHAH, Rafaqat Ali KHAN, Bushra ISMAIL, Asad Mohammad KHAN, Haji MUHAMMAD

**Affiliations:** 1 Department of Chemistry, COMSATS University Islamabad, Abbottabad Pakistan; 2 Department of Chemistry, Federal Urdu University of Arts, Sciences and Technology, Karachi Pakistan

**Keywords:** Antibiotic, cloud point extraction, preconcentration, rifampicin, wastewater

## Abstract

Every year, tuberculosis affects the lungs of millions of people and rifampicin is the commonly used medicine for its treatment due to its antibiotic nature. The frequent use of rifampicin may lead to its increased concentration in the water resources. This research work is focused on the cloud point extraction (CPE) procedure for the preconcentration of rifampicin prior to its determination in water. The UV/vis spectrophotometric method was adapted for the measurement of rifampicin content after the phase separation. Triton-X 100 was used as the nonionic surfactant which contains hydrophilic polyethylene chain feasible for the extraction of analyte. Various analytical parameters that can affect the extraction efficacy were optimized to achieve linearity of the proposed method in the concentration range of 3.54–81.41 mgL^–1^. The Limit of detection and quantification were 1.261 and 4.212 mgL^–^^1^, respectively. The Preconcentration factor was 40 with relative standard deviation (%RSD) of 2.504%. The standard addition methodology was adopted for the validation of this procedure and effectively applied for the determination of rifampicin in real wastewater samples.

## 1. Introduction

Tuberculosis (TB) is a lung infection disease and one of the major causes of death until the end of twentieth century in many countries [1]. TB, like HIV and malaria, has been realized as a great threat for humans all over the world, therefore, in 1993 the World Health Organization declared TB as the great global threat. In 2005, 12 million TB cases were observed and 1.5 million deaths occurred due to TB [2]. For the treatment of TB patients, different antibiotics such as rifampicin and isoniazid have been widely used [3].

Rifampicin (RIF) introduced in 1972 as an antitubercular agent [4], belongs to the special macrocyclic group of antibiotics effective against *Mycobacterium* tuberculosis [5]. Beside this, RIF plays a vital role during sporadic stage to kill semi dormant Tuberclue *bacilli* [6,7]. Due to the presence of the macrocyclic ring in RIF, which is considered as a key structural unit, it is able to interact with the microbial DNA-dependent RNA polymerase and inhibit the introduction of RNA synthesis [8,9]. RIF can be toxic for biological systems and can give rise to several side effects such as, allergic rashes, nausea, hepatotoxicity, appetite loss, immunological disturbances [10], oxidative conjunctivitis [11], fatigue, headache [12], and organic brain syndrome [13].

Anti-TB drugs in different samples have been determined with a number of instrumental techniques such as, high performance liquid chromatography (HPLC) [14], infrared spectroscopy [15], liquid chromatography, and gas chromatography coupled with the mass spectrometry [14]. Liquid chromatography-tandem mass spectrometry for rifampicin determination has been widely used with low detection limit in environmental samples [16]. The traditional extraction techniques used for the extraction and removal of drugs in water samples have some limitations such as time consumption and use of excessive toxic organic solvents (especially, in case of liquid-liquid extraction and solid phase extraction) [17]. Recently, the focus has been shifted towards more economic and efficient techniques requiring the least utilization of solvents, shorter analysis time, as well as maximum preconcentration factor [18].

Increase in the development of surfactant-based extraction methods in sample preparation is observed during the last few decades where the most widely explored surfactant-based preconcentration process is the cloud point extraction (CPE). CPE rely on the phase behavior of nonionic and zwitter ionic surfactants in aqueous solutions which exhibit phase separation upon temperature change or addition of salt [19]. CPE is an eco-friendly process because this technique follows the principles of green chemistry, by utilizing nonvolatile surfactants with less toxicity than those organic solvents [20]. Moreover, CPE has been widely utilized in separation science for extraction, purification, and preconcentration of different analytes prior to their analysis by UV/vis spectrophotometry. The UV/vis spectrophotometry is well-known for its simplicity, reliability, and low cost operation. These features make UV/vis spectrophotometry a good choice against sophisticated detection techniques [20]. Therefore, the aim of this work was to develop a quick, selective, and simple CPE method for the preconcentration of RIF in real wastewater samples.

## 2. Experimental

### 2.1. Reagents and chemicals

Water for experimental work was purified on water distillation apparatus IM-50 (IRMECO GmbH & Co. KG, Schwarzenbek-Germany). The fresh working standard solutions for RIF were prepared on daily basis through stepwise dilution of the stock standard solutions purchased from Merck, Darmstadt, Germany. HPLC grade ethanol was purchased from BDH Laboratory Supplies (England) while sodium chloride was obtained from (Scharlab S.L, Barcelona-Spain). Nonionic surfactant Triton X-100 was obtained from Merck (Darmstadt, Germany) and was used without further purification. A 2.5% (v/v) nonionic surfactant solution was prepared by dissolving 2.5 mL of Triton X-100 (Merck, Darmstadt, Germany) in 100 mL distilled water.

### 2.2. Instrumentation

Absorbance measurements were performed with double beam UV/vis spectrophotometer (PG Instruments Limited, BMS, UK) equipped with deuterium and tungsten lamp. For the pH adjustments, pH meter (Adwa Instruments Kft. Szeged-Hungary) was used. Centrifuge machine (Eppendorf AG, 22331 Hamburg-Germany) was employed for the centrifugation of samples. The constant temperature water bath (Thermostat, DAHAN Scientific, North America, WB-22) was used to maintain temperature required for phase separation.

### 2.3. Samples

Wastewater samples were collected from different places (suburbs and inside the Abbottabad city). The samples were then filtered using a vacuum filter funnel (porosity of 25–50 µm, Aldrich) to remove any suspended particulate matter.

### 2.4. CPE procedure

For CPE preconcentration, the aliquots (20 mL) standard solutions containing 15 mgL^–1 ^RIF (pH = 9) containing 1.0 mL of Triton X-100 (2.5% v/v) were transferred into polypropylene tubes of 50 mL capacity and then heated at constant temperature of 65 °C (5 min). Later to achieve phase separation, the mixture was centrifuged at 3500 rpm for 5 min. Then samples were cooled inside a refrigerator at 0 °C (5 min) to increase the viscosity of Triton X-100, the upper phase was carefully removed by pipette. The viscosity of the coacervated phase was lowered by adding 2 mL ethanol. This helped us to have the required solution volume essential for the UV/vis spectrophotometry. Standard solution of RIF was scanned in visible range of 300–550 nm to find the wavelength (λ_max_) for maximum absorbance as shown in Figure S1. All the measurements were carried out at the constant wavelength (λ_max_) of 338 nm for subsequent analysis.

## 3. Results and discussion

3.1. Optimization of experimental parameters

### 3.1.1. Effect of pH

**Figure 1:**
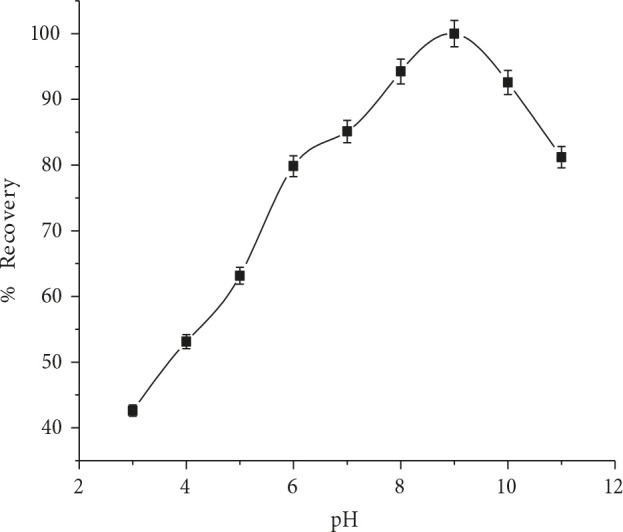
Effect of pH on RIF %recovery (n = 7).

The influence of pH value on speciation of organic compounds in aqueous sample will lead to affect the extraction proficiency. The optimum pH value for RIF recovery were investigated in the range of 3–11, while the other parameters were kept constant. The results showed that appreciable improvement of RIF recovery was observed in the alkaline range (see Figure 1). Then it was decreased at pH greater than 9. The extraction efficiency of RIF decreases in acidic solutions due to the protonation of nitrogen in structure of RIF Figure S2. Therefore, pH 9 is the optimum value selected for the subsequent experiments.

### 3.1.2. Effect of temperature and incubation time

**Figure 2:**
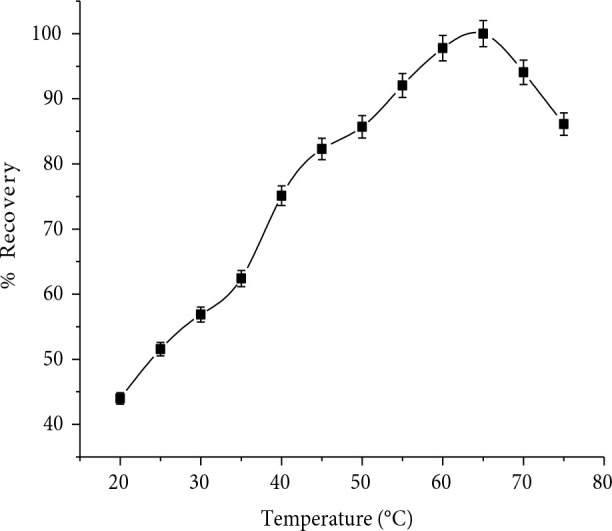
Effect of temperature ( C) on RIF %recovery (n = 7).

Cloudpoint determines the temperature of the phase inversion (clarity/cloudiness). Temperature above the ‘cloud point’ is called cloud point temperature. Therefore, we observed maximum solubilizing ability of analyte for the Triton X-100 at 65 °C due to its 67 °C cloud point temperature [21]. However, it is desirable to employ the lowest possible equilibration temperature and shortest incubation time, to complete the reaction and efficient separation of phases. For better phase separation the temperature parameter was investigated (20–75 °C). The Figure 2, indicates that the highest recovery was obtained at 65 °C, thus this temperature was chosen for subsequent experiment in order to achieve quantitative recovery. 

### 3.1.3. Effect of ionic strength

**Figure 3:**
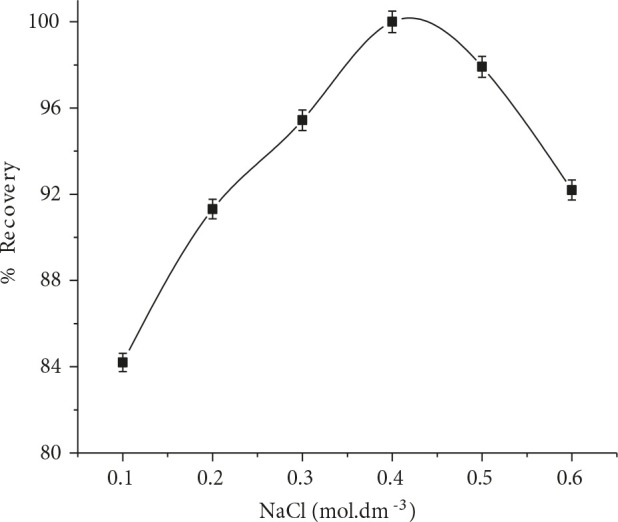
Effect of ionic strength on RIF %recovery (n = 7).

The addition of NaCl into the sample solution can enhance the phase separation phenomenon of aqueous phase and surfactant rich phase. The aggregation and micelle size is enlarged as the concentration of NaCl increased but critical micellar concentration (CMC) remains constant [22]. The effect of NaCl concentration on preconcentration factor and overall recovery was examined in the range of 0.1–0.6 mol.dm^–3^ as illustrated in Figure 3. It appeared that, the extraction efficiency of RIF was quantifiable while using 0.4 mol.dm^–3^. In this way, 0.4 mol.dm^–3^ was utilized in the subsequent experiments.

### 3.1.4. Effect of Triton X-100 concentration

**Figure 4:**
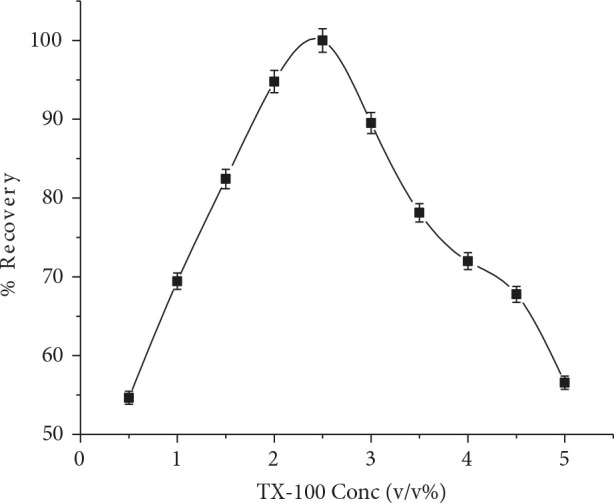
Effect of TX-100 concentration (v/v%) on RIF %recovery (n = 7).

For an effective CPE, the extraction efficiency can be enhanced by minimizing the surfactant phase volume, thereby ensuring the highest preconcentration factor. The usefulness of Triton X-100 concentration in preconcentration efficacy was examined in the range of 0.5%–5% (v/v). The maximum absorbance was observed at 2.5% (v/v). Figure 4 highlights that quantitative recovery was reduced by increasing the surfactant concentration due to the loss of UV/vis spectrophotometer signal. Thus, 2.5% (v/v) concentration was chosen for the subsequent experiments.

### 3.1.5. Effect of sample volume

**Figure 5:**
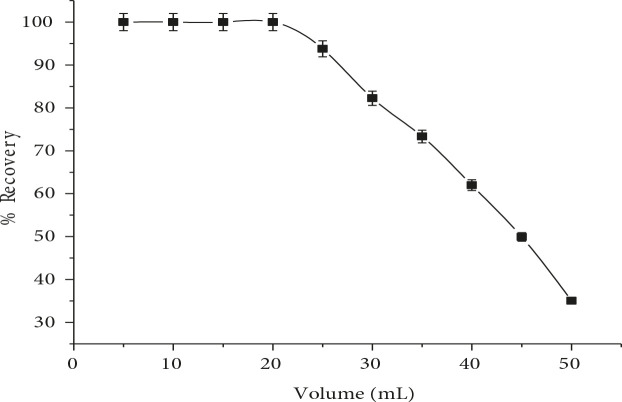
Effect of sample volume on RIF %recovery (n = 7).

The sample volume plays vital role in the preconcentration during the analysis of real samples for obtaining good yield [23]. The effect of sample volume on the extraction efficiency was examined in the range of 5–50 mL. The results Figure 5, showed the extraction efficiency of RIF was almost same up to 20 mL, but reduced with higher sample volumes. The slight decrease in the extraction efficiency is due to insufficient interaction between the analyte and extracting phase. On the basis of these results, 20 mL of sample volume was chosen for subsequent experiments.

### 3.1.6. Effect of centrifugation rate and centrifugation time

**Figure 6:**
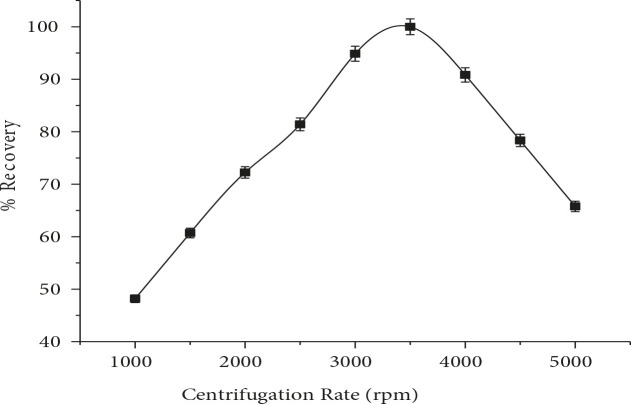
Effect of centrifugation rate on RIF %recovery (n = 7).

**Figure 7:**
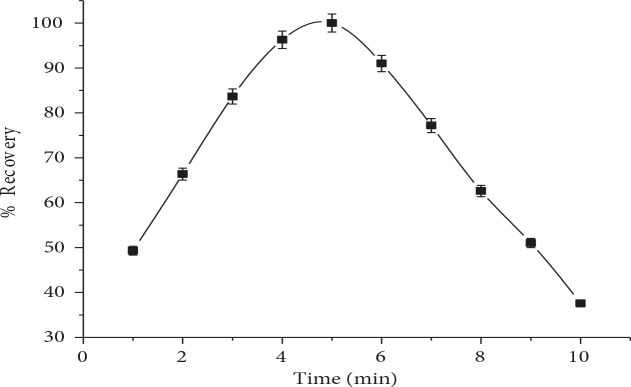
Effect of centrifugation time on RIF %recovery (n = 7).

Centrifugation rate and time affect the efficiency of CPE as these factors play vital role in phase separation of surfactant from aqueous phase [24]. It is aimed to preconcentrate RIF; centrifugation rate was studied in the range of 1000–5000 rpm. The results in Figure 6 indicate that maximum recovery occurs at 3500 rpm and no further improvement was observed for a prolonged period. The influence of the centrifugation time (1–10 min) on CPE of RIF was also examined. The results revealed that significant recovery was observed at 5 min which was selected for further experiments as depicted in Figure 7. 

## 3.2. Interference study

The presence of coexisting species in the real samples and their interference effects are well known in the instrument based analysis [25]. In present study, the tolerable error, which is defined as the effect of interferences on the extraction of RIF was investigated. The maximum number of coexisting species that generate an error not beyond 5% during investigation of RIF are termed as tolerable limit. There can be different interferences, such as isoniazid, pyrazinamide, and ethambutol attributed to the analytical signal of RIF. Stock solutions of 20 mg mL^–1^ of RIF were prepared in the presence of isoniazid, pyrazinamide, and ethambutol and were subjected to the CPE procedure and then finally determined by UV–vis spectrophotometry. Resulting data indicates in Table 1, that no considerable effect of the tested drugs is observed on RIF signals.

**Table 1:** Effect of interfering drugs on the recovery of RIF (n = 7).

Sample no.	Interferences	Tolerance limit(mgL–1)	% Recovery of RIF (150 mgL–1)
1	Pyrazinamide	400	99.1 ± 1.25
2	Ethambutol	275	
3	Isoniazid	75	

## 3.3. Validation of proposed CPE through standard addition method

The standard addition method was used to validate the applicability of proposed method. Real wastewater samples were analyzed after the addition of known quantity of drugs for the determination of RIF through proposed CPE method followed by the UV/vis spectrophotometer. The recoveries of RIF Table 2 were quantifiable for trace analysis and no noticeable matrix was observed.

**Table 2:** Standard addition methodology for the validation of CPE (n = 7).

Sample	Added amount (mg)	Concentration (mgL–1)	Found (mg L–1)	% Recovery
Wastewater	0.0	0.0	BDL	---
	0.2	10	9.89 ± 0.61	98.9 ± 6.11
	0.4	20	19.84 ± 0.73	99.2 ± 7.30

## 3.4. Analytical figures of merit

It is very important to examine the accuracy and precision of the proposed method, therefore, this method was utilized for the preconcentration followed by determination of RIF in the wastewater samples. As illustrated in Table 3, the recoveries of RIF were found reasonable under the optimized experimental conditions. The linear regression, calibration equation, correlation coefficient, limit of detection (LOD), limit of quantification (LOQ), and preconcentration factor are summarized in Table 3. The linear concentration was in the range of 4.12–81.41 mg L^–1^.

**Table 3:** Analytical figures of merit for proposed CPE methodology (n = 7).

Linear range (mgL–1)	4.21–81.41
Calibration equation (mgL–1)	A = 0.0294C + 0.0199
Correlation coefficient (R2)	0.998
Limit of detection (mgL–1)	1.261
Limit of quantification (mgL–1)	4.212
% Relative standard deviation	2.504
Preconcentration factor	40

The extraction recovery ( *ER* ) was calculated according to the following equation:

The *m*_surfactant_ is the concentration of analyte in the final surfactant phase while *m*_aq _is the initial concentration of analyte in the sample solution, *C*_surfactant_, and *C*_aq_ are the analyte concentration in surfactant phase and in the aqueous phase, respectively. *V*_surfactant_ and *V*_aq_ are the concerned volumes of the phases. The LOD and LOQ were calculated by the 3 s/m and 10 s/m, respectively, where s represents standard deviation from 10 blank measurements and *m* is the slope of the calibration curve. The LOD and LOQ obtained were 1.261 and 4.212 mgL^–^^1^, respectively. The preconcentration factor (PF) was calculated as the ratio between the initial volume (sample) and the final volume. 

PF = V_(initial)_/V_(final)._

Excellent analytical figures of merit were obtained with proposed procedure while utilizing simpler instruments (i.e. UV/vis spectrophotometer) and the results can be compared with some literature reported techniques as shown in Table 4 [26–31]. 

**Table 4:** Comparison of the proposed CPE methodology with other reported technique for the determination of RIF.

Sample preparation technique	Detectiontechnique	Sample	Detectionlimit	Linear range	%R.S.D	%Recoveries	Reference
Liquid–liquid extraction	HPLC–UV	Human plasma	-	1–50 mg L–1	15	83	26
		Liver sample	-	0.6–40 µg g–1	15	95	
Glutathione-cappedCdTe/ZnS QDs	Fluorescence	Aqueous solution	0.25 mg mL–1	0.83– 56mg mL–1	-	98.6–103.2	27
Liquid extraction	Reversed phaseHPLC-UV	Human plasma	-	2–20 mg L–1	5 – 23	91.73	28
		Human urine	-	20–200 mg L–1	5 – 23	96.24	
None	HPLC–UV	Pharmaceutical	0.2 mg L–1	1–40 mg L–1	≤ 2.5	99.7–100.5	29
Strata-X-CW extractioncartridge	HPLC–UV	Human plasma	0.15 mg L–1	0.5–20 mgL–1	≤ 8.0	84.5	30
		Human blood spot	1.5 mg L–1			65.0	
Glassy carbon electrode/nanostructured nickel hexacyanoferrate	Voltammetry	Human urine	2.6 µmol L–1	5.0 × 10−6–5.0 ×10−4 mol L–1	4.2	97.0–102	31
CPE	UV/vis spectrophotometry	Wastewater	1.261 mg L–1(1.53 µmol L–1)	4.21–81.41mg L–1	2.504	99.2	Present

## 4. Conclusion

This research demonstrated the optimized, efficient cloud point extraction method for the selective extraction, preconcentration, and detection of RIF from the wastewater samples. The proposed method allowed the determination of minute quantity of RIF in real samples by the UV/vis spectrophotometer, which is available in most of the laboratories. Cloud point extraction via Triton X-100 offered a sensitive, selective, rapid, reproducible, and inexpensive alternative to other separation and preconcentration techniques. The proposed procedure resulted in good precision, accuracy, and better limit of detection. As compared to other separation procedures, this technique is highly eco-friendly. It was observed that the addition of salt is effective in phase separation to prevent the possible loss of analyte during heating.

Supplementary Materials
