# Supplementary figures and images for: Preconcentration of rifampicin prior to its efficient spectroscopic determination in the wastewater samples based on a nonionic surfactant
Source: Turk J Chem. 2021 Aug 27;45(4):1201–9. doi: 10.3906/kim-2102-28 (PMC8517608; doi:10.3906/kim-2102-28)

## Supporting material

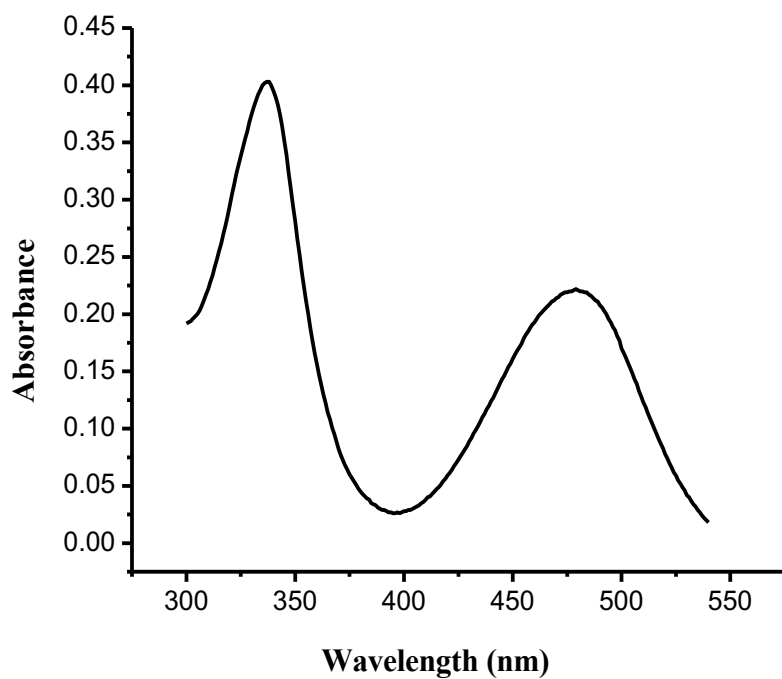

**Figure S1.** Visible absorption spectra for RIF.

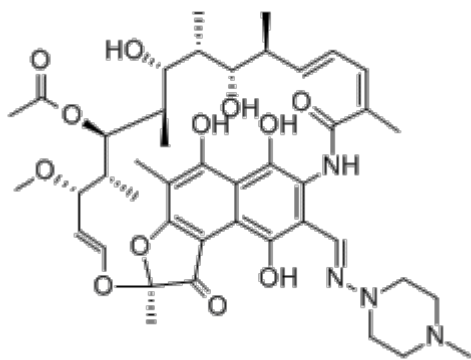

**Figure S2.** Structure of RIF.

Supplement: Supplementary file 1 — Supplementary Materials [file turkjchem-45-1201-sup001.pdf]
